# Analysis of Reciprocally Dysregulated miRNAs in Eutopic Endometrium Is a Promising Approach for Low Invasive Diagnostics of Adenomyosis

**DOI:** 10.3390/diagnostics10100782

**Published:** 2020-10-03

**Authors:** Evgeny Borisov, Margarita Knyazeva, Veronika Novak, Lidia Zabegina, Tatyana Prisyazhnaya, Aleksey Karizkiy, Igor Berlev, Anastasia Malek

**Affiliations:** 1 Subcellular Technology Lab., N.N. Petrov National Medical Research Center of Oncology, 197758 Saint-Petersburg, Russia; evgeny737373@yandex.ru (E.B.); margo9793@gmail.com (M.K.); lidusikza@yandex.ru (L.Z.); iberlev@gmail.com (I.B.); 2 Oncosystem Ltd., 121205 Moscow, Russia; 3 Institute of Biomedical Systems and Biotechnologies, Peter the Great St. Petersburg Polytechnic University, 195251 Saint-Petersburg, Russia; 4 Department of Obstetrics and Gynecology, North-Western State Medical University Named after I.I. Mechnikov, 195067 Saint-Petersburg, Russia; marzhevskayav@inbox.ru (V.N.); tprisyazhnaya@yandex.ru (T.P.); 5 Information Technologies and Programming Faculty, Information Technologies, Mechanics and Optics University, 197101 Saint-Petersburg, Russia; alexei_karitski@mail.ru

**Keywords:** endometriosis, adenomyosis, microRNA, miRNA, two-tailed RT-qPCR, eutopic endometrium

## Abstract

Endometriosis is a chronic disease characterized by the growth of endometrial tissue outside of the uterine cavity. Endometriosis affects up to 10% of women of reproductive age and has great social impact. The diagnostics of endometriosis are based on clinical appearance, ultrasound, and magnetic resonance imaging (MRI); however, a diagnosis is frequently hampered by the absence of objective criteria. Adenomyosis (AM) is a particular type of endometriosis wherein the spread of the ectopic endometrial gland is limited by the uterine myometrium. Alteration of the microRNA expression profile in the eutopic endometrium can be associated with AM, and may be assayed for diagnostic purposes. In the presented study, we aimed to explore the diagnostic potency of this approach. Eutopic endometrium specimens were collected from patients (n = 33) and healthy women (n = 30). The microRNA expression was profiled to select individual microRNAs with AM-associated expression alterations. A new method of two-tailed RT-qPCR microRNA analysis was applied to assay potential markers. The expression ratios of reciprocally dysregulated microRNAs were calculated, and the diagnostic potency of these parameters was evaluated by receiver operation curve (ROC) analysis. Mir-10b, miR-200c and miR-191 were significantly dysregulated in the eutopic endometrium of AM patients. The expression ratio of reciprocally dysregulated microRNAs allowed us to diagnose AM with a range of sensitivity from 65% to 74%, and of specificity from 72% to 86%. The analysis of microRNAs from the eutopic endometrium might present a promising low-invasive method of AM diagnostics.

## 1. Introduction

Endometriosis (EM) is a common gynecological disorder caused by the persistence of dysfunctional endometrial implants outside the uterine cavity, and is associated with a complex of symptoms, including, but not limited to, chronic pelvic pain, dysmenorrhea and subfertility [1]. The variable clinical manifestations of the EM are reflected in the complicated classifications, based on the anatomical distribution and histological structure of the ectopic lesions [2]. Adenomyosis (AM) is a particular condition wherein the detected spread of the ectopic endometrial gland is limited by the uterine myometrium. The overall incidence of EM is estimated at between 6% and 10%, with a high prevalence among young women. The diagnosis of EM is usually hampered by the absence of specific symptoms and still requires empirical approaches. Thus, various combinations of physical examinations, pelvic ultrasounds and MRIs are proposed as first- and second-line diagnostic algorithms by the different national clinical guidelines [3,4,5]. Invasive methods, such as hysteroscopy or laparoscopy, followed by a histopathological exam, are considered exclusively in the severe clinical cases, but cannot be recommended for wide application. Despite practical need, there are no molecular or genetic markers available for the diagnosis of EM as of yet.

The lack of efficient diagnostic tests arises from the still poor understanding of the disease’s pathophysiology. The origin of endometrial-like ectopic implants is still the subject of various hypotheses. They can be classified into two general theories: “in situ development” and “distant dissemination”. In first case, endometrial-like implants are supposed to originate from the local tissue due to incomplete embryonic morphogenesis [6]. In the second case, ectopic endometrial-like glands originate from the eutopic endometrium through various routes of dissemination, for instance, by so-called retrograde menstruation [7]. Some of these hypotheses have only historical interest, while some are still used as a theoretical background for unclear clinical observations [8]. Despite the etiology of EM remaining unclear, great progress in molecular insights regarding the disease’s pathogenesis has been made during recent years [9]. The results of a large number of investigations indicated that the multifactorial nature of EM appears as a result of the complex combination of hormonal, immunological, inflammatory, genetic and epigenetic factors. Careful consideration of these factors may indicate avenues for the development of new approaches for effective and low-invasive diagnostics.

The state of the art of EM biomarker discovery was analyzed recently [10,11]. The authors of these comprehensive reviews tended to classify new biomarkers of EM according to their pathogenic role. Thus, the inflammatory components of EM gave rise to diagnostic methods based on the detection of anti- or pro-inflammatory cytokines in the peritoneal fluid and plasma [12]. EM-associated aberrations of the immune system’s functionality (decreased T cell reactivity and natural killer (NK) cells cytotoxicity, the polyclonal activation of B cells, the production of autoantibodies, and altered neutrophil-to-lymphocyte or lymphocyte-to-monocyte ratios) might be evaluated via liquid biopsy tests [13,14]. Recent investigations revealed the specific EM-associated characteristics of the eutopic endometrium. These may have a direct link to the disease’s pathogenesis, and be of great practical interest due to the availability of biological material. For instance, the altered characteristics of stem cells [15] or the density of nerve fibers [16] in the eutopic endometrium might serve as diagnostic markers of EM. Multiple EM-associated molecular, genetic or epi-genetic characteristics of the eutopic endometrium were described as well. For instance, a number of studies revealed EM-associated alterations of microRNA (miRNA) expression in the eutopic endometrium [17,18,19,20]. 

Thus, miRNA is considered as a promising marker for EM [21]; however, the development and clinical implementation of miRNA-based diagnostic systems are not trivial issues, for two methodological reasons. First, the short length of miRNA molecules and the presence of non-mature forms makes miRNA analysis difficult with conventional reverse transcription followed by qPCR. Second, the absence of a reliable method of RT-qPCR data normalization raises doubts in the results interpretation. In the presented study, we aimed to define the miRNA expression signature of the eutopic endometrium associated with adenomyosis (AM), and to explore the potency of a miRNA-based diagnostic tests. To this end, we (i) applied new highly sensitive technology for miRNA-specific reverse transcription, with a two-tailed primer followed by quantitative PCR, and (ii) explored the concentration ratios of miRNAs with the reciprocal (opposite) character of AM-associated dysregulation as a new diagnostic parameter. Combining these approaches, we developed a new miRNA-based method for a low-invasive diagnosis of AM with relatively high sensitivity and specificity.

## 2. Materials and Methods

### 2.1. Ethical Approval and Study Design

**Figure 1:**
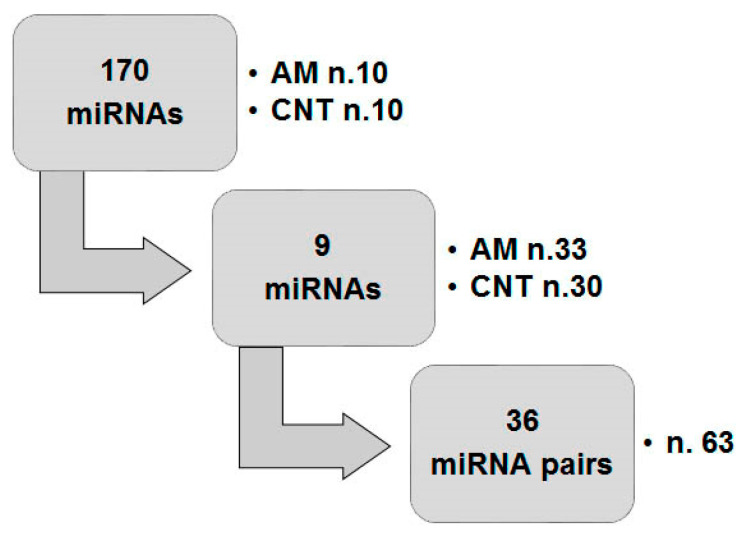
Work flow of the study.

The study was approved by the Local Ethic Committee of the N.N. Petrov National Medical Research Center of Oncology (the project № AAAA-A18-118012390157-2 from 1 November 2017). An informed consent form was signed by each patient. The investigations were performed in two steps. First, the adenomyosis-associated miRNA (n. 170) expression alterations in the eutopic endometrium were preliminarily assayed using pooled samples obtained from patients (adenomyosis, AM), n = 10, and healthy women (control, CNT), n = 10. Then, the differential expression of selected miRNAs was confirmed in individual samples of two groups (patients with AM, n = 33 and healthy women, n = 30). Finally, the concentration ratios of all possible pairs of reciprocally dysregulated miRNAs were calculated and the diagnostic values of these parameters were evaluated by ROC analysis. A schematic design of the study is presented in Figure 1.

### 2.2. Participants

Patients (n = 33) who had been diagnosed with adenomyosis based on complaints, physical examinations, transvaginal ultrasounds (TVUS) and MRIs were recruited to participate in this study. The severity of adenomyosis was defined using TVUS evaluation of location (uteri wall affected), differentiation, presence of adenomyotic cysts, degree of uterine layer involvement, extent of disease and size of lesions (Table 1). Each patient was investigated by two US specialists and their conclusions were well concordant. We used a scoring system largely based on the diagnostic criteria published in 2015 [22] and recently updated by the Morphological Uterus Sonographic Assessment group [23]. This system assumes four levels of assessment that well correlate with clinical status [24,25]. The study included patients with 3–4 degrees of AM severity.

The control group (n = 30) included healthy women. We excluded patients with autoimmune or metabolic disease, pelvic inflammatory disease, myoma, fibroids and dysfunctional uterine bleeding. The average age of participants was 28 (24–35) years. All women were not taking any medication for at least 4 months prior to being involved in the study.

### 2.3. RNA Sampling

Endometrial biopsies were collected using Pipelle suction curettes. Endometrial tissue samples were classified by histological exam, and only patients in the proliferative phase of the cycle (days from the 6th to 13th within the regular menstrual cycle) were included in the study. Tissue collected for miRNA analysis was placed in 1.5 mL of RNA later (sodium citrate 25 mM, EDTA 20 mM, and ammonium sulfate 0.7 g/mL, pH 5.2) and stored at −20 °C. Biological material was pelleted by centrifugation, RNA later was removed and replaced by 600 μL lysis buffer (5M guanidine thiocyanate, 1 M mixture of sodium citrate and citric acid, 10% sodium lauryl sarcosinate, and 14 μM 2-mercaptoethanol) and incubated on a shaker at 65 °C for 15 min. Dense fragments of the tissue were pelleted by centrifugation and the transparent lysate was transferred into the new tube. Afterward, 2-propanol (600 μL) and 10 μL of superparamagnetic particles (Sileks Ltd., Moscow, Russia), were added to the samples, well mixed, incubated on a shaker for 10 min, and centrifuged for 15 min at 18,000× *g*. The supernatant was drained and superparamagnetic particles with adsorbed RNA were washed twice by 70% ethanol (500 μL), then washed with acetone (300 μL) and dried. To elute RNA, the particles were mixed with 1.5 M Tris-HCl, pH 8.8 (300 μL), incubated for 5 min on a shaker at 65 °C, and centrifuged for 2 min at 18,000× *g*. The RNA solutions were replaced in fresh tubes and stored at −80 °C. The concentration and purity of the extracted RNA was analyzed on an Implen NanoPhotometr N60 spectrophotometer (Implen Gmb, Munchen, Germany). The RNA concentrations ranged from 150 to 800 ng/μL. The preparations were considered pure with absorption rates of A260/A280 above 1.8.

### 2.4. miRNA Expression Profiling Using Exiqon microRNA PCR Panel

For the purpose of preliminary miRNA screening, we chose 10 samples from patients with adenomyosis and 10 samples from the control group. Aliquots of individual RNA samples were combined in equivalent quantities into two pools representing adenomyosis (AM) and healthy controls (CNT). In the first step, the RNA was polyadenylated and reverse transcribed using a miRCURY LNA Universal RT microRNA Polyadenylation and cDNA synthesis Kit (Quigen, Hilden, Germany). Then, quantitative PCRs were performed using miRCURY LNA miRNA Focus PCR Panels (kat. N. 339325) and ExiLENT SYBR Green master mix (both from Quigen, Hilden, Germany) on a CFX96 Touch™ Real-Time PCR Detection System (Bio-Rad Laboratories Inc, Hercules, CA, USA). The inter-plate amplification rate discrepancy was corrected with interpolate calibrators. Values of the cycle threshold (Ct) higher than 38 were considered as background and excluded from analysis. The results were normalized to the global Ct mean (28.2).

### 2.5. Quantitative Real-Time RT-PCR (qPCR)

In order to assay the selected miRNA’s (n = 9), two-tailed RT-qPCR (ttRT-qPCR) systems were designed according to the authors recommendation [26]. In contrast to the initial design, supposing the use of dsDNA intercalating agents for real-time detection of amplification, we explored fluorescent (FAM)-labeled probes. Each system was validated with serial dilutions of synthetic miRNAs (mimics). All oligonucleotides were synthesized by Sintol Ltd., Moscow, Russia. The IDs of miRNAs, the sequences of the miRNA synthetic mimics, the primers and the probes are presented in Supplementary Table S1. The CFX96 Touch™ Real-Time PCR Detection System (Bio-Rad Laboratories Inc, Hercules, CA, USA) was used for all reactions. Reverse transcription (RT) was performed using 1 μL RNA solution and 1 μM of ttRT primes with the M-MuLV-RH RT kit (Biolabmix Ltd., Novosibirsk, Russia) in 20 μL reaction volume. The reaction was conducted at 25 °C for 45 min, followed by incubation at 85 °C for 5 min to inactivate reverse transcriptase. Quantitative PCR was performed using a 2 μL RT reaction mix, PCR primers (6 μM both), and FAM-labeled probe 4 μM with the BioMaster HS-qPCR (2×) kit (Biolabmix Ltd., Novosibirsk, Russia). The conditions for qPCR were as follows: 95 °C for 10 min and 45 cycles of 95 °C for 5 s followed by 65 °C for 15 s. Finally, a melting curve analysis was performed. U6 snRNA was used as the endogenous control. The RT-qPCR reactions for each miRNA molecule were repeated in triplicate and averaged. The results of RT-qPCR were normalized to the total mean of Ct and to the Ct of the reference (U6 snRNA) independently. The CFX Manager Software, SigmaPlot 11.0 and GraphPad Prism 8.0.2 software were used for the data handling and analysis. 

### 2.6. Calculation and Statistics

The statistical significance of the observed difference in the miRNA expression between the two groups (AM vs. CNT) was evaluated using the Mann–Whitney test, assuming a non-parametric distribution of the analyzed parameter.

The selection of the optimal “reciprocal miRNAs pair” with high diagnostic potency (two miRNAs that have opposite AM-associated expression alterations) was done by testing all possible pairs of dysregulated miRNAs. Reciprocal pairs were formed as all possible combinations of the selected set of miRNAs. The total number of analyzed pairs could be estimated as $P_{n}^{r}=\frac{n!}{\left(n-r\right)!}$ (the number of ordered sets of miRNAs of size R, without replacement, from the total set of examined miRNAs of size N). For every pair, amplification ratios (ratio = $2^{Ct\left(miR-A\right)–Ct\left(miR-B\right)}$) were calculated and used as a discriminative marker to distinguish the AM and CNT samples. As the total number of pairs could be too large for manual analysis, the tasks of pair forming, calculating amplification ratios, and the receiver operation curve analysis were performed programmatically. The results obtained from the ROC analysis were sorted via the area under the curve (AUC) values.

## 3. Results

### 3.1. Preliminary miRNA Profiling

**Figure 2:**
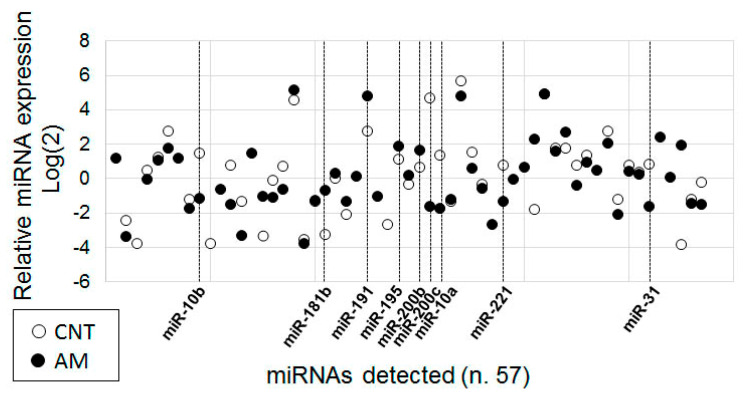
The miRNA expression profile of the eutopic endometrium of healthy women (CNT) and women with adenomyosis (AM). The results of the RT-PCR analysis of the pooled samples: 10 RNA samples from the endometrium of healthy women (CNT) and 10 RNA samples from the ectopic endometrium of women with adenomyosis (AM). The PCR results were normalized to the global Ct mean (28.2) and log_2_ transformed. The expression in either CNT or AM samples was detected for 57 miRNAs from the 170 tested. miRNAs with obvious expression differences between CNT and AM are indicated.

In total, 10 samples of RNA isolated from the eutopic endometrium of women diagnosed with adenomyosis were combined in equivalent quantities into one pool (AM). The control pool (CNT) was also composed of 10 samples of RNA isolated from the endometrium of healthy women. In each pool, the expression of the 170 miRNAs was assayed using polyadenylation followed by universal RT and miRNA-specific qPCR. Interplate calibrators (in four plates) resulted in almost equal Ct values (from 21.23 to 21.35), indicating equivalent RNA quality and PCR efficacy between the plates. Values of Ct higher than 38 were considered as background and excluded from the analysis. After correction of the inter-plate discrepancy, the results were normalized to the global Ct mean (28.2), log_2_ transformed, and presented in Supplementary Table S2 and as a scatter plot (Figure 2). The total number of the detected miRNAs in the CNT pooled sample was 45, while the total number of miRNAs detected in the AM pool was 54. The expression level of 26 molecules was measured in both samples. We selected nine miRNAs for further evaluation: four miRNAs that were up-regulated (miR-181a, miR-191, miR-195 and miR-200b) and five miRNAs that were down-regulated (miR-10b, miR-200c, miR-10a, miR-221 and miR-31) in the AM pool compared to the CNT pool. Selection was based on several criteria, as follows: the degree and direction of AM-associated expression alterations, and the structure of mature miRNA that allowed for the confident design of a two-tailed RT-qPCR analytic system. The selected miRNAs are indicated in Figure 2.

### 3.2. Creation and Validation of ttRT-qPCR Systems for Individual miRNA Analysis

The systems for miRNAs analysis, composed of a two-tailed reverse transcription (ttRT) primer and two miRNA-specific PCR primers, were designed according to the recommendations of P. Androvic et al. [26]. In order to increase the specificity of the method, we used FAM-labeled probes instead of the DNA binding dye SYBR Green. The analytic properties of the ttRT-qPCR systems were evaluated using synthetic analogues (mimics) of the corresponding miRNAs.

**Figure 3:**
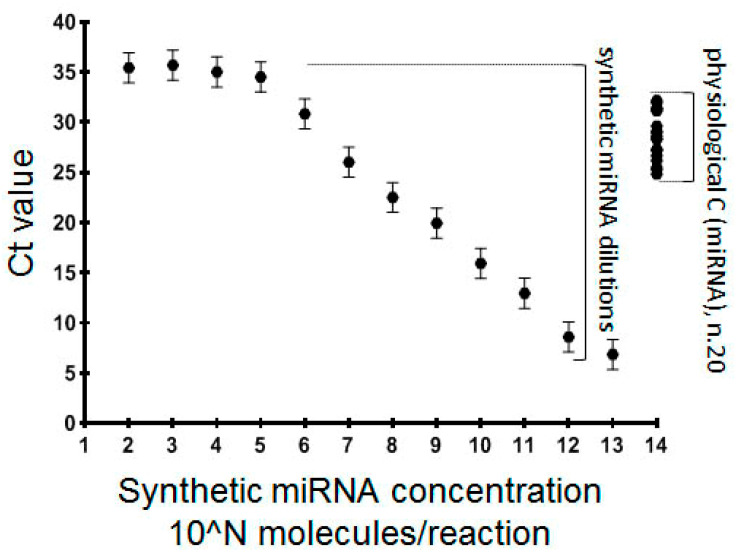
Analytic properties of the tt-RT-PCR system for miRNA-10b. The system (tt-RT-PCR) for miR-10b analysis was designed and tested with a row of synthetic mimic dilutions from 10^2^ to 10^13^ molecules per RT reaction. A linear dependency between the miR-10b concentration and the Ct value was observed in a range 10^5^ to 10^12^. The physiological concentration of miR-10b in a collection of 20 RNA samples was tested additionally.

The mimics were diluted in a broad range of concentrations and used in amounts from 10^2^ to 10^13^ molecules per reaction of reverse transcription. PCR was performed in triplicate and the Ct values of the technical replicas were averaged. Representative results of the dependency of amplification rate (threshold cycle, Ct) on miRNA-mimic concentration for the miRNA-10b system are presented in Figure 3. The area of the linear dependency of the Ct value on the miRNA concentration was observed in a range of 10^5^–10^12^ molecules per reaction. For other systems, the minimal measured miRNA concentration varied between 10^4^ and 10^7^ molecules/reaction. With the further decrease of the mimic concentration down to zero, constant values of Ct were observed. Thus, different RT-qPCR systems reached their analytic “plateau” at different Ct values (Table 2).

We did not observe any amplification in the reaction mix without the RT primer (data not shown). This observation indicates the possibility that the amplification started at low concentrations, or even the absence of target miRNAs due to interactions between the RT primer (50–56 nt. length) and the partially complementary PCR primers. This technical feature might be critical in cases of the low physiological concentration of miRNA. To explore whether the analytic sensitivity of created systems would be sufficient to measure the corresponding molecules in endometrium specimens, each of the 20 randomly selected samples were analyzed using all nine ttRT-qPCR systems. Representative results (for miR-10b) are plotted in the graph (Figure 3), and the other results are in Table 2. As the physiological concentration of all miRNA molecules was measured within the area of the linear dependency of the Ct value on the synthetic miRNA concentration, the analytic properties of all systems were deemed sufficient to be used in further analysis.

### 3.3. Analysis of Selected miRNAs by ttRT-qPCR Systems

**Figure 4:**
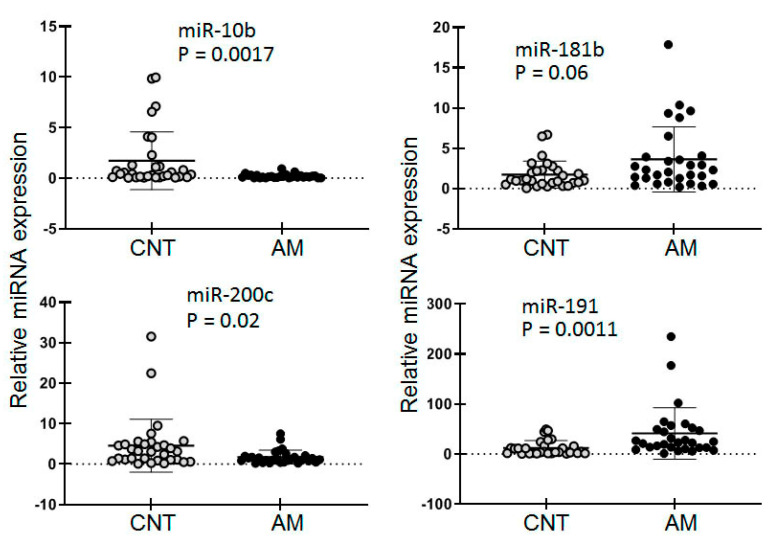
The expression of selected miRNAs in the eutopic endometrium of healthy women (CNT, n = 33) and women with adenomyosis (AM, n = 30) tested by tt-RT-PCR systems. The results were normalized to the U6 snRNA and the relative expression data were grouped (CNT and AM). The statistical significance of the miRNA expression difference between the groups was estimated using the nonparametric Mann–Whitney test.

The expression levels of nine selected miRNAs were analyzed in 30 samples of RNA isolated from the endometrium of healthy women, and 33 samples of women with AM. All measurements were performed in triplicate, and the results were averaged and normalized to the global Ct mean (Ct averaged for 567 measurements) and to the Ct of snRNA U6 for each individual sample. To compare the AM and CNT groups, we calculated the mean of the normalized results and the standard error of the mean (mean +/− SEM) for each miRNA after the two methods of normalization. Concordant results were observed for six of the nine miRNAs tested. For three miRNAs (miR-10a, miR-31, and miR-221), different results in the comparison of the AM vs. CNT groups were achieved after the different methods of normalization. This indicated the high impact of the normalization approach. The results obtained by normalization to the Ct mean are presented Table 3. The high variability of the results within each group was expected due to the likely complex factors, including the natural spread of the miRNA’s activity in the population and in the endometrium during the menstrual cycle. To estimate the selected miRNA expression difference between the groups (AM vs. CNT), we applied the nonparametric Mann–Whitney test. With this approach, a statistically significant expression difference (AM vs. CNT) was detected for miR-10b, miR-191 and miR-200c (Figure 4). We tested the diagnostic potency of these molecules using ROC analysis with 63 samples (AM, n = 33 and CNT, n = 30); the AUC value did not reach 0.65 in any case.

The obtained results revealed the low diagnostic potency of single miRNA, which indicated the need to either to identify new, more effective markers (i), or to develop a new method for data analysis (ii). We explored the latter approach.

### 3.4. Identification of Reciprocally Dysregulated miRNA Pairs and Analysis of Their Diagnostic Potency

The absence of reliable reference (“house-keeping”) miRNA that can be used for normalization of the miRNA RT-qPCR results is the known problem. In the case of a large miRNA profiling study, using the averaged (global) Ct values of multiple measurements as the normalizer was proposed and might be helpful [27,28,29]. However, this approach is hardly applicable for small data sets that are intended to be implemented as diagnostic tests for clinical use. In several previous studies [30,31], we reported the utility of alternative methods of miRNA expression data analysis. We demonstrated that the ratio of amplification rates, calculated as 2^Ct(miR-A)-Ct(miR-B)^, for two miRNAs with opposite (reciprocal) characteristics of disease-associated expression alteration, might serve as a good diagnostic marker. In the cited above study focused on cervical cancer, the selection of such miRNAs pairs was easy because of the clearly staged disease progression, from normal status, through low- and high-grade squamous interepithelial lesion, to cervical cancer. Two miRNAs that had gradual increases and decreases in expression alteration might compose a “reciprocal pair” with high diagnostic potency.

**Figure 5:**
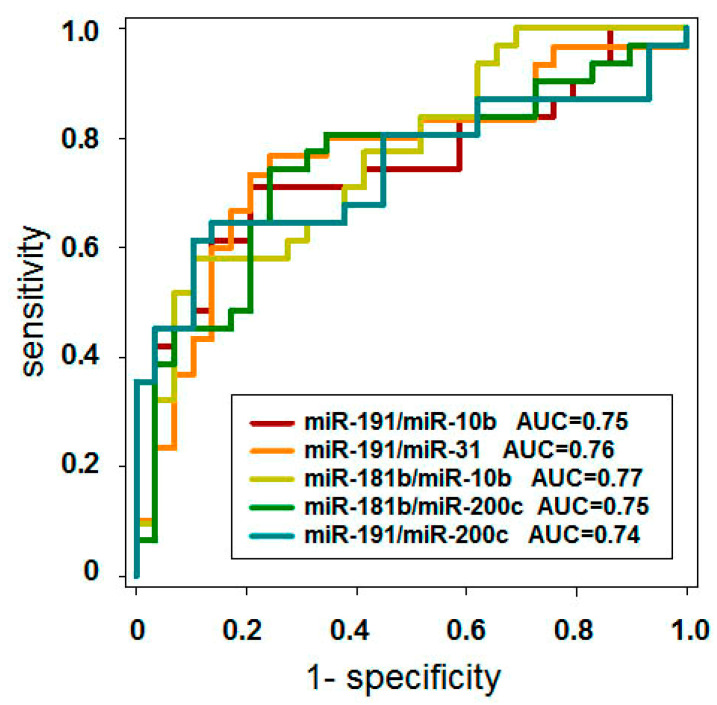
Receiver operation curve (ROC) analysis of the ratios of reciprocally dysregulated miRNA pairs. The concentration ratios of reciprocally dysregulated miRNAs pairs were calculated and the diagnostic potency of these parameters was estimated by ROC analysis with set of 63 samples (CNT, n = 33 and AM, n = 30). Areas under curve (AUC) values (A) are shown.

In the case of a simple diagnostic dilemma such as “AM vs. CNT”, the selection of an optimal reciprocal miRNA pair can be done by testing multiple random pairs of dysregulated miRNAs. In the present study, we tested all possible combinations of nine miRNAs in terms of their diagnostic potency by ROC analysis using a group of 63 cases (AM, n = 33 and CNT n = 30). The best five results are presented in Figure 5 and Table 4. Thus, the expression ratio of miR-181b/miR-10b allowed us to distinguish AM from healthy controls (CNT) with quite high accuracy; AUC = 0.77 (sensitivity 61.29 and specificity 72.41). Our results do not provide any indication of whether the observed phenomenon has any biological basis or is only a random event. This requires further investigation. However, even without biological rationale, the proposed approach can solve the problem of expression data normalization, and can be utilized for the development new miRNA-based diagnostic methods.

## 4. Discussion

The presented study was performed with a limited number of biological samples (AM, n = 33 and CNT, n = 30) and the miRNAs profiling was limited to 170 molecules only. However, the obtained results are supported by published data; the identified miRNAs have been already reported to be implemented in endometriosis development. For instance, miR-191 was found to be over-expressed in endometriomas tissue and to be a key regulator in the proliferation and the invasive properties of endometriosis cells (CRL7566) in vitro [32]. MiR-181b is involved in the regulation of the endometrial stromal cells’ migratory capacity [33]. The effect of miR-10b down-regulation in the endometrial cells may be realized via Syndecan-1, resulting in increased invasiveness [34]. The experimental inhibition of miR-200c in the primary endometrial stromal cells promoted the proliferation and migration in vitro, while the local delivery of the miR-200c mimic inhibited the growth of ectopic endometrial lesions in vivo [35]. These results support our observations of the AM-associated up-regulation of miR-191 and miR-181b, and the down-regulation of miR-10b and miR-200c. Thus, even with limited input data, our approach allowed us to identify highly potential markers of AM, confirmed by independent reports.

Our study was planned to explore new approaches to analyze miRNA expression (i), and to interpret the miRNA expression data (ii) with the general purpose of developing an miRNA-based test for the low-invasive diagnosis of AM. We optimized and explored the method of miRNA reverse transcription (RT) proposed by P. Androvic [26]. According to the author’s reports, the technology of two-tailed priming of RT followed by PCR with two miRNA-specific primers provided exceptional specificity of analysis. Within the presented study, we demonstrated that the sensitivity of ttRT-qPCR was also sufficient for the analysis of marker miRNA in the endometrium. However, all our ttRT-qPCR systems reached their sensitivity “plateau” when the concentration of synthetic miRNA dropped down to 10^4^–10^7^ molecules/reaction (Figure 3 and Table 2). We presume this “plateau” was the result of the interaction of the ttTR primer and PCR primers, because no amplification was observed in the absence of the RT mix in PCR (data not shown). These were model experiments with pure solutions of analyzed mimics. When real biological samples are analyzed, the presence of other types of RNAs would likely reduce the interactions of the primers. Taking this technological aspect into account, we suggest the necessity of proving that the physiological concentration of the tested miRNA is measured in the range of the linear ratio of Ct value to miRNA concentration obtained by the ttRT-qPCR system. Otherwise, in cases of low concentrations of the analyzed miRNA in biological samples, this “plateau” of amplification signal could be mistakenly interpreted as positive results. This may limit the applicability of the proposed technology for low-copy miRNAs.

The development and the clinical implementation of the miRNA-based diagnostic tests is challenged by the absence of a reliable approach for data normalization expression. In contrast to the well-established method of gene-coding RNA normalization vs. the mRNA level of “house-keeping” genes, miRNAs’ expression patterns vary considerably in different tissues, and “house-keeping” miRNAs are barely identified. For example, miR-16 was validated as a normalizer for cervical cytology samples [36], miR-151a-3p, -197-3p, -99a-5p, and -214-3p for thyroid biopsy [37], miR-520d, -1228, and -345 for colon cancer tissue [38], miR-24, -103a, and let-7a for lymph nodes [39] and mir-152 and -23b for the liver [40]. The information regarding possible miRNA normalizers in circulating plasma or serum is also discordant. The concentration of small nuclear or small nucleolar RNAs (snRNA or snoRNA) is suggested to be relatively constant, and these molecules are widely used for the normalization of miRNAs expression data. For instance, the combination of RNU48, U75 and RNU44 was proposed as a confident normalizer for endometrial tissue [41]. However, comparative studies revealed quite different levels of snRNA and snoRNA expression stability, which also depends on the method of analysis [42]. Different alternative strategies, such as the calculation of the geometrical mean of several exogeneous miRNAs [43] and the combination or global average of endogenous miRNA [28], were proposed. This indicates the lack of any universally accepted normalization strategy.

In previous studies, we demonstrated the efficacy of the new approach based on the assumption that the ratio of the concentrations of two miRNAs with opposite (reciprocal) characteristics of disease-associated expression change might have a higher diagnostic potency compared to individual molecules [30]. This phenomenon may have a biological ground if the functions of two miRNAs are linked with the regulation of disease-relevant cellular properties, but are opposing. In such cases, reciprocal expression shifts might become more significant with disease progression. As our understanding of the complex system of miRNA-mediated gene expression control remains insufficient to identify or even predict reciprocally dysregulated miRNAs pairs, we tested all possible miRNA combinations. This “mechanistic” approach allowed us to identify several pairs of miRNAs in which the expression ratio had diagnostic values much higher than any of these molecules estimated individually (0.74–0.77 vs. 0.5–0.65). Further research and a deeper understanding of the role of miRNAs in the pathogenesis of AM will likely disclose the biological meaning of the bi-directional dysregulation of certain molecules, and will allow us to identify miRNAs pairs with higher diagnostic potency.

The clinical utility of the described approach will be defined by the most important parameters of diagnostic accuracy, specificity and sensitivity. The specificity achieved by proposed reciprocally dysregulated miRNAs pairs can be assumed as satisfactory. However, the sensitivity is obviously not enough for clinical utilization. The combination of relatively high specificity (60–97%) and low sensitivity (57–87%) is reported also for commonly used TVUS [44]. Thus, a highly sensitive complimentary diagnostic method is especially required. There are two ways to increase the sensitivity of the miRNA-based test: the identification of new miRNAs pairs with robust AM-associated reciprocal dysregulation, and the increase of the number of such pairs included in the diagnostic test. Both approaches require additional research involving genome-scale miRNAs profiling.

## 5. Conclusions

Conclusively, AM is associated with specific alterations of the miRNA profile of the eutopic endometrium. Analysis of the miRNAs in the eutopic endometrium may present a new, promising method for the low-invasive diagnosis of AM. Considering the accessibility of the biopsy and the relatively low cost of PCR, the proposed approach merits further investigation. After additional scientific conformation and technological optimization, this method can be proposed for implementation in routine gynecological practice.

## Figures and Tables

**Table 1:** Adenomyosis (AM) group characteristics: TVUS-assessed items.

Location (Uteri Wall)	Anterior = 4	Posterior = 22		Mixed/Other = 7
Differentiation	Focal = 10	Diffuse = 17		Adenomyoma = 6
Cystic	Yes = 28		No = 5	
Uterine layer involvement	Type 1–2 = 19	Type 2–3 = 8		Type 1–3 = 6
Extent of adenomyosis	Moderate = 21		Severe = 12	
Size of lesions	3–6 cm = 8	7–9 cm = 22		10 and >=3

Numbers reflect number of patients with corresponding characteristics among group of 33 participants.

**Table 2:** Analytic properties of the two-tailed RT-qPCR system for miRNA detection.

miRNA	Method Sensitivity Limit for Synthetic miRNA		Results of miRNA Analysis in Biological Samples(Ct Value Range and Median)
	Plateau of Ct Value	Minimal Concentration (Molecules/Reaction)	
-10a	36.3	10^4^	29.32–33.41 (31.80)
-10b	34.7	10^5^	28.12–31.97 (30.60)
-31	30.4	10^5^	26.03–28.78 (27.45)
-181b	29.5	10^7^	27.03–28.03 (27.45)
-191	31.4	10^5^	21.42–25.44 (23.67)
-195	32.5	10^5^	25.31–29.12 (27.31)
-200b	33.4	10^5^	24.11–25.25 (26.87)
-200c	33.7	10^5^	25.18–30.22 (27.53)
-221	32.6	10^6^	29.55–31.88 (30.88)

**Table 3:** Results of the comparison of selected miRNAs expression in groups of samples.

miRNA-		-10a	-10b	-31	-181b	-191	-195	-200b	-200c	-221
CNT	Mean	0.23	1.72	3.49	2.38	20.04	7.56	3.48	4.57	0.43
	SEM	0.06	0.51	0.76	0.71	6.18	2.77	0.93	1.17	0.14
AM	Mean	0.24	0.19	3.05	3.63	41.34	8.48	5.71	1.73	0.22
	SEM	0.06	0.04	0.55	0.75	9.56	2.16	3.02	0.32	0.07
Mann–Whitney		NS	0.0017	0.9490	0.0680	0.0011	NS	NS	0.0200	NS

CNT—control group of healthy women (n = 33), patients with adenomyosis (n = 30), Mean is calculated as sum of the normalized expression rates divided by the number of values (CNT, n = 33 and AM, CNT = 30), SEM is standard error of the means.

**Table 4:** The diagnostic potency of reciprocally dysregulated miRNA pairs.

	miR-191/-10b	miR-191/-31	miR-181b/-10b	miR-181b/200c	miR-191/-200c
Diagnostic potency (AUC)	0.75	0.76	0.77	0.75	0.74
Sensitivity (%)	70.97	73.33	61.29	74.19	64.52
Specificity (%)	79.31	79.31	72.41	75.86	86.21

## Supplementary Materials

The following are available online at https://www.mdpi.com/2075-4418/10/10/782/s1, Table S1: Sequences of oligonucleotides (5’-3’) used for RT-qPCR analysis (two-tailed RT-qPCR system). Table S2: Results of miRNAs profiling in pooled samples of RNA isolated from eutopic endometrium of healthy women (CNT, n = 10) and patients with AM (AM, n = 10).
