# Supplementary material for: Analysis of Reciprocally Dysregulated miRNAs in Eutopic Endometrium Is a Promising Approach for Low Invasive Diagnostics of Adenomyosis
Source: Diagnostics (Basel). 2020 Oct 3;10(10):782. doi: 10.3390/diagnostics10100782 (PMC7601074; doi:10.3390/diagnostics10100782)
Supplement: Supplementary file 1 [file diagnostics-10-00782-s001.zip › Borisov_Suppl data 2.docx]

Borisov E. et all., Analysis of reciprocally dysregulated miRNAs in eutopic endometrium is a promising approach for minimally invasive diagnostics of adenomyosis

Supplementary data 2.

**Results of miRNAs profiling in pooled samples of CNT (eutopic endometrium of healthy women) and AM (eutopic endometrium of patients with AM). Data are normalized to averaged Ct and Log2 transformed. MiRNAs marked were selected for further analysis.**

|  |  | CNT | AM |
| --- | --- | --- | --- |
| 1 | hsa -let-7a-5p |  | 1,21 |
| 2 | hsa-miR-103a-3p | -2,43 | -3,33 |
| 3 | hsa-miR-106b-5p | -3,78 |  |
| 4 | hsa-miR-107 | 0,49 | -0,01 |
| 5 | hsa-miR -125b-5p | 1,26 | 1,08 |
| 6 | hsa-miR-126-3p | 2,78 | 1,78 |
| 7 | hsa-miR-130a-3p |  | 1,19 |
| 8 | hsa-miR-132-3p | -1,2 | -1,7 |
| 9 | hsa-miR-10b-5p | 1,48 | -1,14 |
| 10 | hsa-miR-141-3p | -3,77 |  |
| 11 | hsa-miR-143-3p |  | -0,61 |
| 12 | hsa-miR-145-5p | 0,78 | -1,51 |
| 13 | hsa-miR-146a-5p | -1,33 | -3,3 |
| 14 | hsa-miR-26a-5p |  | 1,5 |
| 15 | hsa-miR-150-5p | -3,27 | -1,03 |
| 16 | hsa-miR-155-5p | -0,1 | -1,1 |
| 17 | hsa-miR-15b-5p | 0,7 | -0,61 |
| 18 | hsa-miR-16-5p | 4,6 | 5,16 |
| 19 | hsa-miR-17-5p | -3,5 | -3,76 |
| 20 | hsa-miR-181a-5p | -1,33 | -1,24 |
| 21 | hsa-miR-181b-5p | -3,26 | -0,68 |
| 22 | hsa-miR-182-5p | 0,04 | 0,34 |
| 23 | hsa-miR-27a-3p | -2,09 | -1,32 |
| 24 | hsa-miR-18a-5p |  | 0,15 |
| 25 | hsa-miR-191-5p | 2,78 | 4,78 |
| 26 | hsa-miR-192-5p |  | -1,01 |
| 27 | hsa-miR-194-5p | -2,67 |  |
| 28 | hsa-miR-195-5p | 1,13 | 1,88 |
| 29 | hsa-miR-196a-5p | -0,32 | 0,19 |
| 30 | hsa-miR-200b-3p | 0,68 | 1,64 |
| 31 | hsa-miR-200c-3p | 4,69 | -1,62 |
| 32 | hsa-miR-10a-5p | 1,35 | -1,72 |
| 33 | hsa-miR-20a-5p | -1,32 | -1,19 |
| 34 | hsa-miR-21-5p | 5,666 | 4,83 |
| 35 | hsa-miR-214-3p | 1,57 | 0,61 |
| 36 | hsa-miR-215-5p | -0,33 | -0,55 |
| 37 | hsa-miR-22-3p |  | -2,68 |
| 38 | hsa-miR-221-3p | 0,8 | -1,32 |
| 39 | hsa-miR-222-3p |  | -0,05 |
| 40 | hsa-miR-223-3p |  | 0,69 |
| 41 | hsa-miR-23a-3p | -1,76 | 2,28 |
| 42 | hsa-miR-23b-3p | 4,9 | 4,9 |
| 43 | hsa-miR-24-3p | 1,78 | 1,63 |
| 44 | hsa-miR-25-3p | 1,78 | 2,72 |
| 45 | hsa-miR-26b-5p | 0,78 | -0,4 |
| 46 | hsa-miR-27b-3p | 1,36 | 0,99 |
| 47 | hsa-miR-29a-3p |  | 0,48 |
| 48 | hsa-miR-29b-3p | 2,78 | 2,06 |
| 49 | hsa-miR-30b-5p | -1,22 | -2,06 |
| 50 | hsa-miR-30c-5p | 0,78 | 0,42 |
| 51 | hsa-miR-30d-5p | 0,38 | 0,25 |
| 52 | hsa-miR-31-5p | 0,87 | -1,62 |
| 53 | hsa-miR-9-5p |  | 2,44 |
| 54 | hsa-let-7e-5p |  | 0,06 |
| 55 | hsa-miR-92b-3p | -3,8 | 1,96 |
| 56 | hsa-miR-93-5p | -1,22 | -1,43 |
| 57 | hsa-miR-99a-5p | -0,22 | -1,48 |
