# Supplementary material for: Analysis of Reciprocally Dysregulated miRNAs in Eutopic Endometrium Is a Promising Approach for Low Invasive Diagnostics of Adenomyosis
Source: Diagnostics (Basel). 2020 Oct 3;10(10):782. doi: 10.3390/diagnostics10100782 (PMC7601074; doi:10.3390/diagnostics10100782)
Supplement: Supplementary file 1 [file diagnostics-10-00782-s001.zip › Borisov_Suppl data 1.docx]

Borisov E. et all., Analysis of reciprocally dysregulated miRNAs in eutopic endometrium is a promising approach for minimally invasive diagnostics of adenomyosis

Supplementary data 1.

**Sequences of oligonucleotides (5’ - 3’) used for RT-qPCR analysis (two-tailed RT-qPCR system)**

| hsa-miR-10a-5p  MI0000266 | Synthetic miRNA | UACCCUGUAGAUCCGAAUUUGUG |
| --- | --- | --- |
|  | Primer RT | ACAGGGTAGATATGTGAGACGTACGTTGAGTACGTCAAGTGAAGTAAATTC |
|  | PCR-forward | CACAGGGTAGATATGTGAGAC |
|  | PCR-reverse | CGTACCCTGTAGATCCGA |
|  | Probe | FAM-ACGTACGTTGAGTACGTCAAGTG-BHQ1 |
| hsa-miR-10b-5p  MI0000267 | Synthetic miRNA | UACCCUGUAGAACCGAAUUUGUG |
|  | Primer RT | ACAGGGTAGATATGTGAGACGTACGTTGAGTACGTCAAGTGAAGTGAAGTAAATTC |
|  | PCR-forward | ACAGGGTAGATATGTGAGAC |
|  | PCR-reverse | CTACCCTGTAGAACCGAA |
|  | Probe | FAM-ACGTACGTTGAGTACGTCAAGTG-BHQ1 |
|  | Synthetic miRNA | AGGCAAGAUGCUGGCAUAGCU |
|  | primer RT | TCTTGCCTCAACGACCAGAGCTAGAGAACCTAGCTCACCCACTACAGCTAT |
|  | PCR-forward | TCTTGCCTCAACGACCA |
|  | PCR-reverse | CAGGCAAGATGCTGGC |
|  | Probe | FAM-AGAGAACCTAGCTCACCCACTAC-BHQ1 |
| hsa-miR-181b-5p  MI0000270 | Synthetic miRNA | AACAUUCAUUGCUGUCGGUGGGU |
|  | primer RT | TGAATGTTGATATGTGAGACGTACGTTGAGTACGTCAAGTGAAGTACCCAC |
|  | PCR-forward | GCTGAATGTTGATATGTGAGAC |
|  | PCR-reverse | CAACATTCATTGCTGTCGG |
|  | Probe | FAM-ACGTACGTTGAGTACGTCAAGTG-BHQ1 |
| hsa-miR-191-5p  MI0000465 | Synthetic miRNA | CAACGGAAUCCCAAAAGCAGCUG |
|  | primer RT | TTCCGTTGCTATGCTCTCCAGGTACAGTTGGTACCTGTCTCCACTTCTGCTT |
|  | PCR-forward | TTCCGTTGCTATGCTCTC |
|  | PCR-reverse | CGCAACGGAATCCCAA |
|  | Probe | FAM-TACAGTTGGTACCTGTCTCCACTT-BHQ1 |
| hsa-miR-195-5p  MI0000489 | Synthetic miRNA | UAGCAGCACAGAAAUAUUGGC |
|  | primer RT | TGCTGCTACAACGACCAGAGCTAGAGAACCTAGCTCACCCACTACGCCAAT |
|  | PCR-forward | TGCTGCTACAACGACCA |
|  | PCR-reverse | CCGTAGCAGCACAGAAATAT |
|  | Probe | FAM-AGAGAACCTAGCTCACCCACTAC-BHQ1 |
| hsa-miR-200b-3p  MI0000342 | Synthetic miRNA | UAAUACUGCCUGGUAAUGAUGA |
|  | primer RT | CAGTATTATCAAGCTCTCCAGGTACAGTTGGTACCTGACTCCACGCTCATCA |
|  | PCR-forward | GCAGTATTATCAAGCTCTCCAG |
|  | PCR-reverse | GCGTAATACTGCCTGGTAATG |
|  | Probe | FAM-ACAGTTGGTACCTGACTCCACGC-BHQ1 |
| hsa-miR-200c-3p  MI0000650 | Synthetic miRNA | UAAUACUGCCGGGUAAUGAUGGA |
|  | primer RT | CAGTATTATCAAGCTCTCCAGGTACAGTTGGTACCTGACTCCACGCTCCATC |
|  | PCR-forward | CAGTATTATCAAGCTCTCCAG |
|  | PCR-reverse | CTAATACTGCCGGGTAATG |
|  | Probe | FAM-ACAGTTGGTACCTGACTCCACGC-BHQ1 |
| hsa-miR-221-3p  MI0000298 | Synthetic miRNA | AGCUACAUUGUCUGCUGGGUUUC |
|  | primer RT | ATGTAGCTCAACGACCAGAGCTAGAGAACCTAGCTCACCCACTACGAAACC |
|  | PCR-forward | ATGTAGCTCAACGACCAGA |
|  | PCR-reverse | CGAGCTACATTGTCTGCTG |
|  | Probe | FAM-AGAGAACCTAGCTCACCCACTAC-BHQ1 |
